# Crystallization of DNA-coated colloids

**DOI:** 10.1038/ncomms8253

**Published:** 2015-06-16

**Authors:** Yu Wang, Yufeng Wang, Xiaolong Zheng, Étienne Ducrot, Jeremy S. Yodh, Marcus Weck, David J. Pine

**Affiliations:** 1 Department of Chemistry, Molecular Design Institute, New York University, New York, New York 10003, USA; 2 Department of Physics, Center for Soft Matter Research, New York University, New York, New York 10003, USA; 3 Department of Chemical & Biomolecular Engineering, Polytechnic School of Engineering, New York University, Brooklyn, New York 11201, USA

## Abstract

DNA-coated colloids hold great promise for self-assembly of programmed heterogeneous microstructures, provided they not only bind when cooled below their melting temperature, but also rearrange so that aggregated particles can anneal into the structure that minimizes the free energy. Unfortunately, DNA-coated colloids generally collide and stick forming kinetically arrested random aggregates when the thickness of the DNA coating is much smaller than the particles. Here we report DNA-coated colloids that can rearrange and anneal, thus enabling the growth of large colloidal crystals from a wide range of micrometre-sized DNA-coated colloids for the first time. The kinetics of aggregation, crystallization and defect formation are followed in real time. The crystallization rate exhibits the familiar maximum for intermediate temperature quenches observed in metallic alloys, but over a temperature range smaller by two orders of magnitude, owing to the highly temperature-sensitive diffusion between aggregated DNA-coated colloids.

## Abstract (web summary)

DNA-coated colloids have failed to achieve their promise of programmable self-assembly because they stick to each other like Velcro. Here Wang *et al.* overcome this problem by making clickable smooth colloids that are coated with short single-stranded DNA at high density.

## Graphical abstract

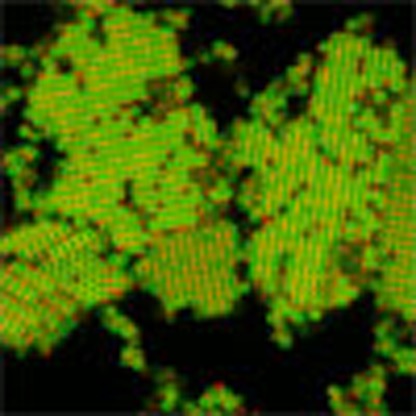

The programmable self-assembly of DNA-coated nanoparticles^1^,^2^,^3^,^4^,^5^,^6^,^7^ has become a well-developed science, complete with a robust set of design principles^5^,^6^. By functionalizing nanoparticle surfaces with different combinations of complementary DNA, an almost limitless variety of colloidal structures can, in principle, be programmed^8^,^9^. However, assembling larger micrometre-sized DNA-coated colloids into three-dimensional crystals has proven much more difficult^10^,^11^,^12^,^13^,^14^, and thus is much less well developed in spite of optical applications that require larger particles^15^,^16^ and the obvious advantages of being able to study the crystallization kinetics by direct observation using an optical microscope^17^,^18^.

The principal impediment has been that (micrometre-sized) DNA-coated colloids condense into random aggregates, but do not crystallize^10^. In some cases, small crystallites form if the particles are smaller than a few hundred nanometres^14^,^19^. More generally, however, when two or more DNA-coated particles bind, they have difficulty rolling over each other and become kinetically trapped. Thus, there is little if any relative diffusion between bound particles, which leads to the formation of random aggregates that are unable to anneal into crystals^10^,^20^. A number of factors have been cited as contributing to the difficulty for bound DNA-coated colloids to diffuse so that they can anneal and form crystalline structures—the inhomogeneity of the interaction potential due to the random relatively sparse distribution of DNA strands on a colloid^21^,^22^, the roughness of the colloid surface^20^ and low areal density of DNA bound to the colloid surface^23^.

Here we show that these problems can be addressed by fabricating particles with a high grafting density of single-stranded DNA (ssDNA), 5 to 25 times greater than previously reported^10^,^22^,^23^, smooth surfaces and short DNA sticky ends, with as few as four bases. These factors enable bound particles to roll over each other near the DNA melting temperature so that the particles can find their free energy minimum and thus form very large crystals. The ability to crystallize micrometre-sized colloids allows us to follow the formation of crystals in real space and time. We observe different crystallization mechanisms, including nucleation and growth as well as spinodal decomposition, with kinetics spanning over a decade in time depending on the quench depth. We then show how the crystallization kinetics is controlled by the sensitive temperature dependence of the surface diffusion of colloids bound by hybridized DNA. We are also able to follow the annealing process, including how various kinds of defects form.

## Results

### Synthesis of DNA-coated microsphere

**Figure 1:**
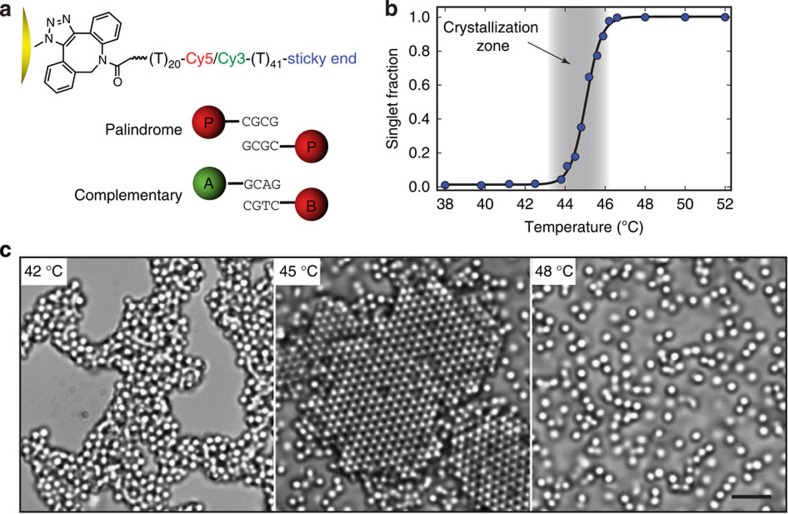
Phase behaviour of DNA-coated micrometre-sized particles. (**a**) Schematic illustration of DNA-coated particles. The DNA is grafted to the particle surfaces using an SPAAC. A 61-base long ‘poly-T' part, internally labelled with a Cy5 (red fluorescence) or Cy3 (green fluorescence) dye, serves as a flexible spacer. We label particles with CGCG (palindrome) sticky ends and Cy5 dye as ‘P', particles with GCAG as sticky ends and Cy3 dye as ‘A' and particles with CTGC as sticky ends (complementary to A) and Cy5 dye as ‘B'. (**b**) Singlet particle fraction (unbound particles) as a function of temperature. Particles are allowed to equilibrate at each temperature for 15 min before a measurement. The temperature window over which particle crystallization occurs is shaded. (**c**) Bright-field optical images showing the morphologies of 1.0-μm P particles—amorphous (left, 42 °C), crystalline (middle, 45 °C) and unbound (right, 48 °C). Scale bar, 5 μm.

While we ultimately employ colloids made from a variety of materials, including poly(styrene), poly(methyl methacrylate) and silica, we focus our discussion on DNA-coated colloids made from 3-(trimethoxysilyl)propyl methacrylate (TPM). We use fluorescently labelled ssDNA consisting of 61-base long ‘poly-T' as a flexible spacer and a short sticky end at the 3′ terminus to provide specific binding to complementary strands via DNA hybridization. The DNA is covalently grafted to the particle surfaces at the 5′ terminus using a strain-promoted alkyne-azide cycloaddition reaction (SPAAC)^24^ (Fig. 1a, see Methods). Each 1.0-μm particle is functionalized with about 115,000 DNA strands (see Methods), which is equivalent to one DNA per 27 nm^2^, a DNA coverage about one or more orders of magnitude greater than previously reported in the literaturere^10^,^22^,^23^. Under a confocal microscope, the particles exhibit a bright and homogeneous fluorescent corona indicating a dense uniform DNA layer (Supplementary Fig. 1).

A collection of DNA-functionalized particles with different sizes and sticky ends are prepared. Particles functionalized with 4-base GCAG sticky ends and Cy3 dye (green) are called ‘A', particles with 4-base CTGC sticky ends that are complementary to A and Cy5 dye (red) are called ‘B' and particles with 4-base CGCG sticky ends that are self-complementary (palindrome) and Cy5 dye are called ‘P' (Fig. 1a).

### Crystallization

**Figure 2:**
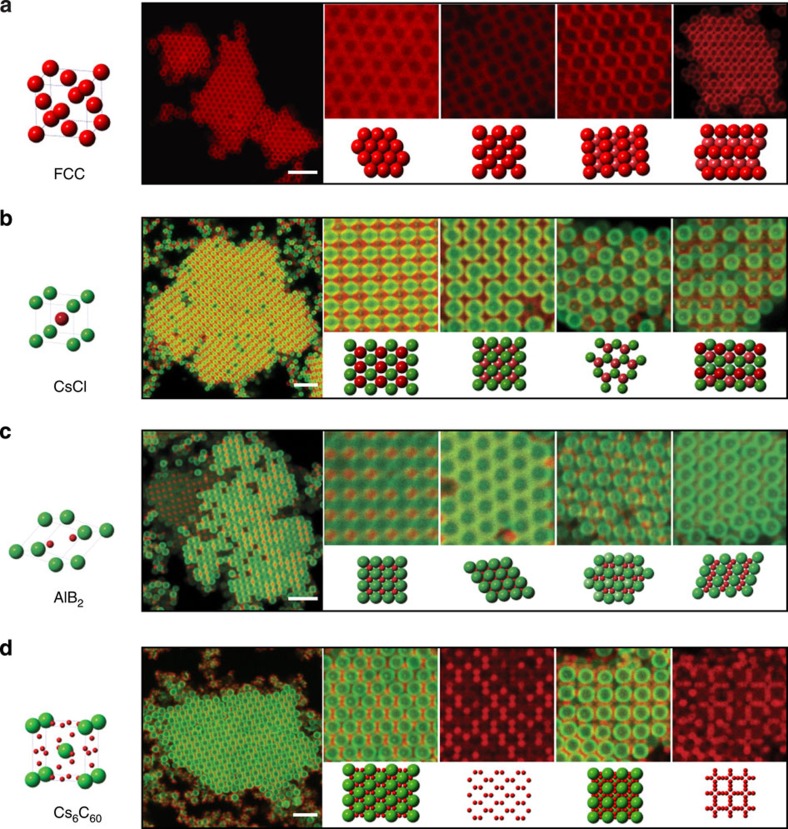
Colloidal crystals assembled from DNA-coated colloids. Confocal fluorescent images and corresponding drawings showing various crystals fabricated from DNA-coated colloids of different sizes and stoichiometries. (**a**) 1.0-μm P particles form face-centered cubic lattice, whose 111, 100, 110 and 311 planes are displayed. (**b**) An AB lattice (isostructural to CsCl) assembled from 1.0-μm A and 1.0-μm B particles with complementary DNA sticky ends. Multiple lattice planes are observed including, from left to right, 110, 100, 111 and 211 planes. (**c**) An AB_2_ crystal (isostructural to AlB_2_) is obtained using 1.0-μm A and 0.54-μm B particles. 100, 001, 111 and 101 planes are shown. (**d**) An AB_6_ crystal lattice (isostructural to Cs_6_C_60_) assembled from 1.5-μm A and 0.54-μm B particles. 110 and 100 planes are shown, accompanied by the corresponding red channel showing only the structural arrangement for the 0.54-μm particles. Interestingly, we find that the AB_6_ structure can tolerate a large number of B vacancies, up to 50%, at the surface of the crystals, while in the bulk there appear to be far fewer. Scale bar, 5 μm.

Particles coated with complementary DNA sticky ends reversibly associate and disassociate on cooling below and heating above a certain melting temperature *T*_m_ (Fig. 1b,c). For 1.0-μm P particles, *T*_m_=46.5 °C. When quenched from above *T*_m_ to *T*=45 °C, crystals spontaneously nucleate after about 5 min and grow to encompass almost the entire sample within 60 min (Fig. 1c, middle panel; see also Supplementary Movie 1). Similar crystallization behaviour is observed for all particle diameters investigated—3.5, 2.0, 1.5, 1.0 and 0.54 μm (Fig. 2a, see also Supplementary Figs 2 and 3a). The crystals extend into three dimensions with the thickness depending primarily on the initial concentration of particles and the thermal gravitational height. We note that in the limit we work in, where the DNA is much shorter than the particle diameter, particles coated with double-stranded DNA never crystallize, whereas particles coated with ssDNA always crystallize for shallow quenches (see discussion of crystallization kinetics below).

We also investigate binary systems formed from A and B colloids bearing complementary sticky ends. For A and B particles of equal size, AB colloidal crystals isostructural to caesium chloride (CsCl) are obtained (Fig. 2b, Supplementary Fig. 3b). Confocal fluorescent images of different crystal planes verify the structures.

By changing the size ratio and stoichiometry of the A and B particles (see Methods), we obtain AB_2_ colloidal crystals isostructural to aluminium boride (AlB_2_; Fig. 2c, Supplementary Fig. 3c) or AB_6_ crystals isostructural to caesium fullerene-complex Cs_6_C_60_ (Fig. 2d, Supplementary Fig. 3d). Figure 2c shows the 100, 001, 111 and 101 planes of the AB_2_ crystal formed using 1.0-μm A and 0.54-μm B particles (size ratio ∼0.54). The images in Fig. 2d show the 110 plane and the 100 plane of an AB_6_ crystal formed using 1.5-μm A and 0.54-μm B particles (size ratio ∼0.36). The structures we observe are consistent with those observed for similar size ratios for oppositely charged spheres^25^,^26^,^27^ and double-stranded DNA-coated gold nanoparticles, but where the DNA strand lengths are comparable to the nanoparticle diameters^5^.

### Mobility of bound DNA-coated colloids

**Figure 3:**
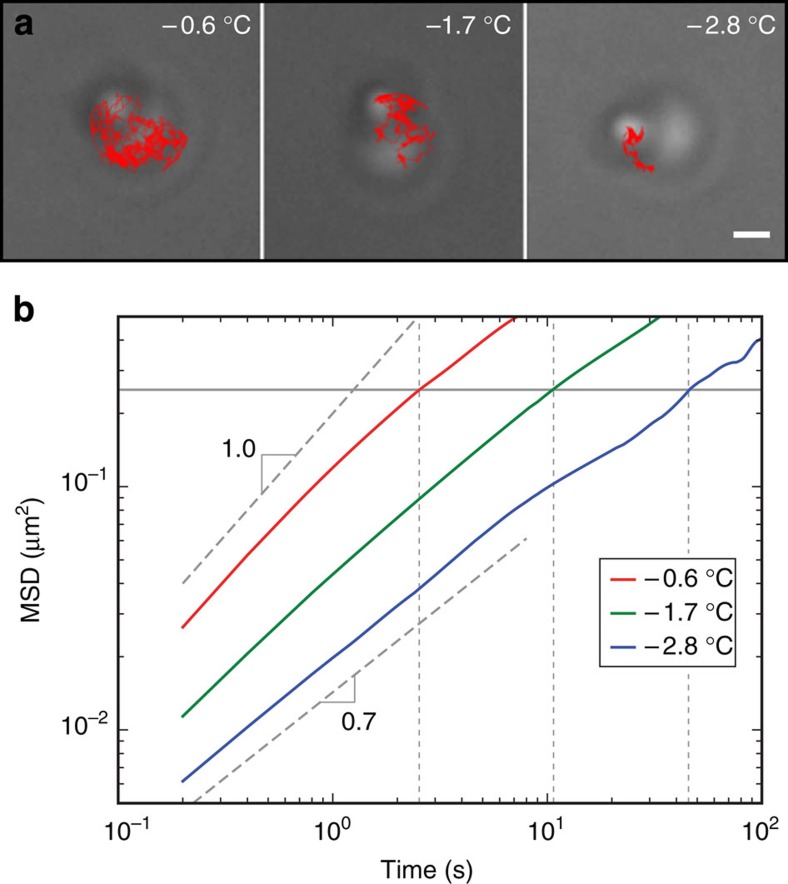
Mobility of bound DNA-coated colloids. (**a**) Bright-field microscope images showing 1.0-μm B particles bounded via DNA hybridization to A particles. The red lines represent the tracks B particles explore in 6 min on A particles at various quench depths relative to the melting temperature; the surface area explored by a particle in a set period of time increases as the temperature increases. The mean square displacement 〈*r*^2^〉 is calculated from those tracks. (**b**) Mean square displacement 〈*r*^2^〉 of a particle rolling on a sphere at three different temperatures. The data are described by 〈*r*^2^〉=*At*^*α*^ where 0.7<*α*≤1. Scale bar, 1 μm.

The ability to roll and rearrange is critical for maximizing the DNA hybridization between bound particles and for the formation of crystalline structures. To investigate the mobility of bound DNA-coated colloids, we track the motion of a 1.0-μm B particle as it diffuses on an immobilized 2.0-μm A particle (Fig. 3a, Supplementary Fig. 4, Supplementary Movie 2, Methods). We find that the mean square displacement 〈*r*^2^〉 of a particle on a sphere is well described by 〈*r*^2^〉=*At*^*α*^ where 0.7<*α*≤1, as shown in Fig. 3b. For temperatures just below *T*_m_, *α* is very nearly 1, which corresponds to normal diffusive motion. For lower temperatures, *α*<1, which means that the particle motion is subdiffusive. This can occur when there is a random distribution of traps, which we expect from random fluctuations in DNA grafting density on the colloids^21^,^22^.

After binding to each other, particles need not diffuse far to crystallize. Assuming the typical distance to be of the order of a particle radius *R*, the characteristic time *τ* can be read off the horizontal grey line at 〈*r*^2^〉=*R*^2^=0.25 μm^2^ in Fig. 3b for each data set. This time increases rapidly with quench depth: *τ*=2.5, 11 and 46 s for quench depths of −0.6, −1.7 and −2.8 °C, respectively. This decrease in particle mobility is due, in part, to the fact that particles diffuse more slowly when their binding energy increases as the temperature is lowered^28^. The decrease is exacerbated by the fact that the particle motion becomes increasingly subdiffusive—α decreases—as the temperature is lowered.

### Crystallization kinetics

**Figure 4:**
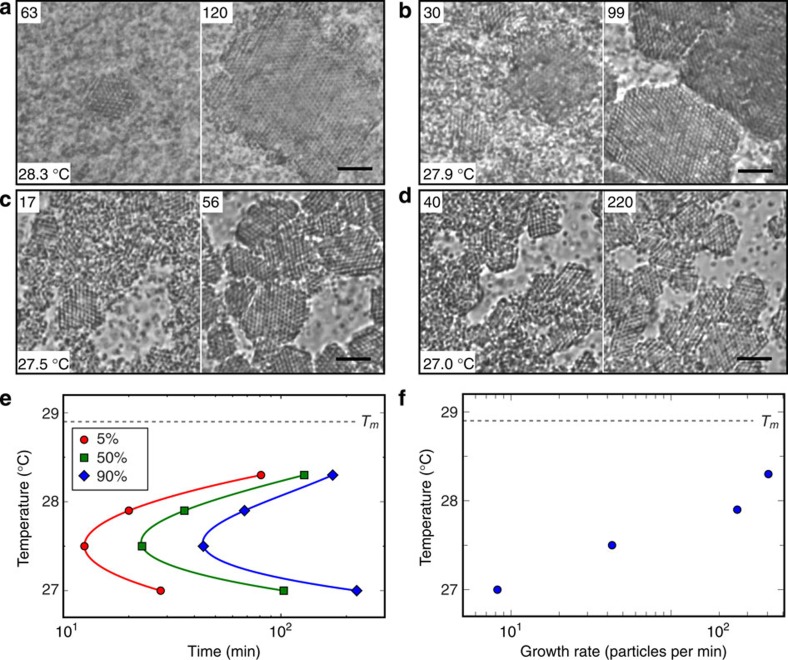
Crystallization kinetics of DNA-coated colloids at various temperatures. Snapshots from Supplementary Movies 3–6 showing the nucleation and crystal growth process of a CsCl lattice assembled from 0.54-μm A particles and 0.54-μm B particles at (**a**) 28.3 °C, (**b**) 27.9 °C, (**c**) 27.5 °C and (**d**) 27.0 °C. The melting temperature is 28.9 °C. The time in minutes after quench is indicated at the upper left of each snapshot. (**e**) Time-temperature-transformation diagram of the CsCl lattice showing the time required for 5, 50 and 90% crystal conversion at four temperatures. The ‘C' shape indicates that the nucleation and overall transformation rate is maximized at intermediate temperatures and decreases when the temperature is either lowered or raised. The connecting lines are for illustration purposes only. (**f**) Diagram showing that the crystal growth rate increases as temperature increases. The growth rate is measured as the average number of particles added to crystal per minute in the observation plane after nucleation.

Crystal formation kinetics depend sensitively on the quench depth. Supplementary Movies 3–6 and Fig. 4a–d show the formation of CsCl crystals at different annealing temperatures. At an intermediate temperature (27.5 °C), it takes only 45 min to crystallize most of the particles (>90%). At higher temperatures (28.3 and 27.9 °C) and at a lower temperature (27.0 °C), the time required to reach 90% conversion is significantly longer. These results are summarized in Fig. 4e where we plot the annealing temperature versus the time required to attain 5, 50 and 90% crystal conversion—the time-temperature-transformation diagram—for the CsCl structure. The curves display the same characteristic ‘C' shape observed in metallic alloys, where the nucleation and overall transformation rate exhibit a maximum for an intermediate temperature quench^29^.

For the shallowest quenches, crystal formation proceeds by nucleation and growth. A time-lapse video (Supplementary Movie 3) at an annealing temperature of 28.3 °C (*T*−*T*_m_=−0.6 °C) reveals incipient clusters of up to six to eight particles across that form and fade over a period of minutes, indicating that the system is metastable. Eventually, a larger crystalline cluster appears after about 45 min (00:45:00), some 15 particles across and grows. As it grows, other incipient clusters appear and fade away. Eventually, some five large separate crystals nucleate and grow to encompass nearly all the available particles (03:00:00) in the field of view.

At 27.9 °C, crystal formation proceeds by a similar process, although the overall transformation time is significantly shorter than for the shallower quench to 28.3 °C, due to a much faster nucleation rate (see Supplementary Movie 4). This is consistent with classical nucleation theory, in which both the free energy barrier and the critical nucleus size decrease as the quench depth is increased^30^. Once nuclei form, the crystals actually grow more slowly than they do for the shallower quench, as revealed by the data in Fig. 4f. We attribute this to the slower rate at which particles diffuse and roll to find their lattice positions after binding to a crystal. Because crystals nucleate so much faster, many more nucleate, which makes their ultimate sizes much smaller than observed for the shallower quench (Supplementary Fig. 5).

For the deepest quenches, crystal formation proceeds by a two-stage process in which a dense amorphous aggregate forms very rapidly, followed by slow crystallization. When the system is quenched to 27.0 °C (*T*−*T*_m_=−1.9 °C), density fluctuations appear almost immediately on length scales much larger than the particle size (00:02:40 in Supplementary Movie 6), which suggests that the system is globally unstable and undergoes spinodal decomposition^31^. Very soon thereafter, a dense metastable amorphous network forms. Subsequently, particles in the network rearrange locally as small crystals form and grow throughout the sample, which results in a large polycrystalline aggregate consisting of approximately a hundred crystallites in the field of view. Here crystals grow by local rearrangements that occur by diffusion, which is very slow for these deep quenches. So even though the first crystals appear very early after the quench, as indicated by the 5% conversion time in the time-temperature-transformation diagram in Fig. 4e, the system proceeds to 90% conversion much more slowly than it does for shallower quenches. This is also reflected in the very slow crystal growth rate, 0.8 particle min^−1^ shown in Fig. 4f for the deepest quench. At 27.5 °C, phase separation also proceeds by the two-stage process of spinodal decomposition to an amorphous aggregate followed by crystallization via local rearrangements (see Supplementary Movie 5). However, particle diffusion within the amorphous aggregate is much faster than it is for the deepest quench discussed above. Thus, the overall time for crystallization is shortest for this intermediate quench.

The time for the initial aggregates to form, whether they are ordered or amorphous, decreases with increasing quench depth (Supplementary Table 1). On the other hand, the time it takes for crystals to grow gets steadily longer as diffusion slows with increasing quench depth. The net result is that the overall crystal formation occurs fastest for intermediate quenches (Fig. 4f). This is similar to what is observed in metallic alloys, where the crystallization rates change over temperature scales of several hundred degrees^28^. Here by contrast, the changes occur over a few degrees.

The crystal formation process for the FCC, AlB_2_ and Cs_6_C_60_ exhibits a similar temperature dependence trend but proceeds at different rates. Palindrome particles crystallize fastest, forming an FCC lattice. For example, 0.54-μm P particles nucleate within 5 min, and 90% conversion is achieved in 15 min at an intermediate quench temperature. The nucleation rate of AlB_2_ and Cs_6_C_60_ is slowed as each particle can bind only with a fraction of the population. Increasing particle size also slows crystallization as larger particles have slower diffusion rates. Increasing the number of bases in the DNA sticky ends slows the crystallization kinetics. For example, changing the length of the sticky ends of DNA on 0.54-μm particles from four (CGCG) to eight (CGTATACG) bases, both palindrome sequences, increases the time required for 90% conversion at intermediate temperature quenches by a factor of four.

### DNA areal density and surface smoothness

**Figure 5:**
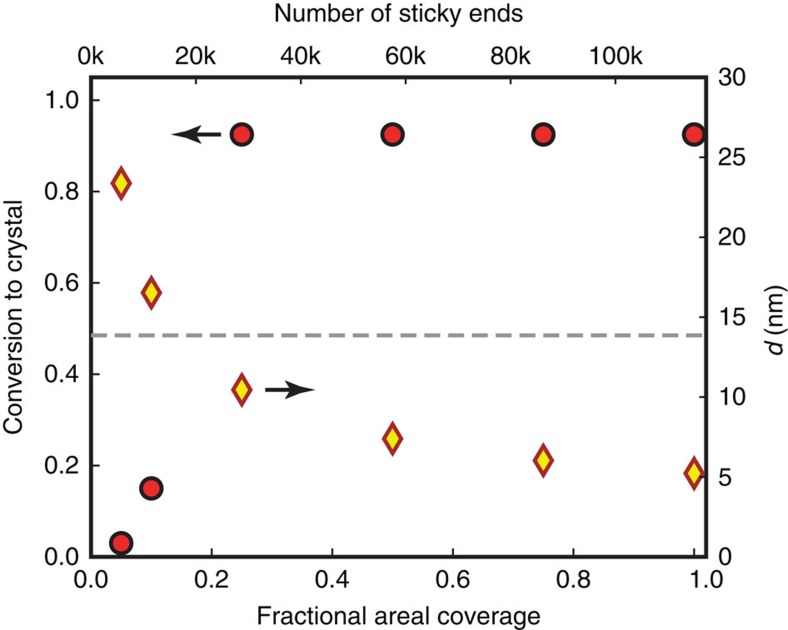
Effect of sticky-end DNA coverage on crystallization. The percentage of DNA-coated colloids converted to crystals after 10 h of annealing (red circles) and the mean distance *d* (yellow diamonds, range from 5 to 22 nm) between grafted DNA sticky end is plotted as a function of sticky-ended DNA surface coverage. Uncertainties are comparable to the size of the data points. The lateral reach *R*_l_ of the 61-base DNA is ∼14 nm (see Supplementary Note 1), as indicated by a dashed grey line. When the DNA coverage is below 10%, *d* exceeds *R*_l_. In this limit, particles fail to completely crystallize. Scale bar, 5 μm.

To investigate the dependence of crystallization on the density of sticky ends on the colloids, we prepare different sets of 1.0-μm TPM particles where the particles are fully functionalized with DNA, but only a fraction of the DNA has sticky ends. The remainder of the DNA has the same 61-base poly-T sequence but lacks the sticky ends. In all cases, the total number of DNA strands per particle remains constant at 115,000; only the fraction of sticky ends changes. Here we use 8-base palindrome sticky ends and find that all the samples crystallize after 10 h of annealing when the fractional coverage is equal to or greater than 25%, or about 28,000 sticky-end DNA strands per particle. When the fractional coverage is 10%, or about 11,500 sticky-end DNA strands per particle, only about 15% of the particles form crystals; at 5% coverage, particles still aggregate but only about 3% form crystals. These data are summarized in Fig. 5.

The lateral reach and the areal density of the grafted sticky-end ssDNA ends determine how many potential partners a sticky end can have on an adjacent colloid. Using literature values for the distance between nucleotides *b*_0_=0.63 nm and the persistence length *L*_p_=2.5 nm (ref. 32; for 100 mM NaCl), the distribution of end heights is about 13–17 nm for our 61-base ssDNA strands, depending on the exact choice for the excluded volume parameter (see Supplementary Note 1)^33^. Assuming the lateral reach *R*_l_ of the DNA ends is Gaussian distributed, 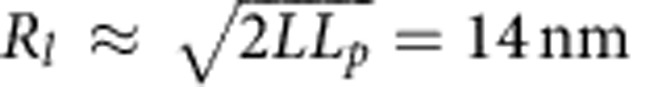, where *L*=*Nb*_0_=38 nm (see Supplementary Equation within Supplementary Note 1). Figure 5 plots the mean distance *d* between active sticky ends and reveals that the particles fail to completely crystallize when *d* exceeds *R*_l_. In this limit, ssDNA strands with sticky ends cease to be able to reach more than one complementary sticky end on the bound particle, which suppresses particles from rolling on each other. This suggests that it might also be interesting to study the effect of the length of the DNA strands on crystallization.

To investigate the effect of surface morphology on crystallization, we prepare 1.0-μm TPM particles with rough surfaces (see Methods and Supplementary Fig. 1b). The root mean square fluctuations in surface height for the rough particles are measured with atomic force microscopy to be 2.0±0.5 nm, as compared with the smooth particles with root mean square height fluctuations of <0.5 nm (Supplementary Fig. 1d). The particles are functionalized with sticky-ended DNA (CGCG), with a DNA surface coverage of 9.1 × 10^4^ DNA per particle. After repeated annealing near the melting temperature for 24 h, no crystalline structure is observed, in contrast to particles prepared with smooth surfaces. We speculate that surface roughness creates local free energy minima with large barriers to rolling.

### Defects

**Figure 6:**
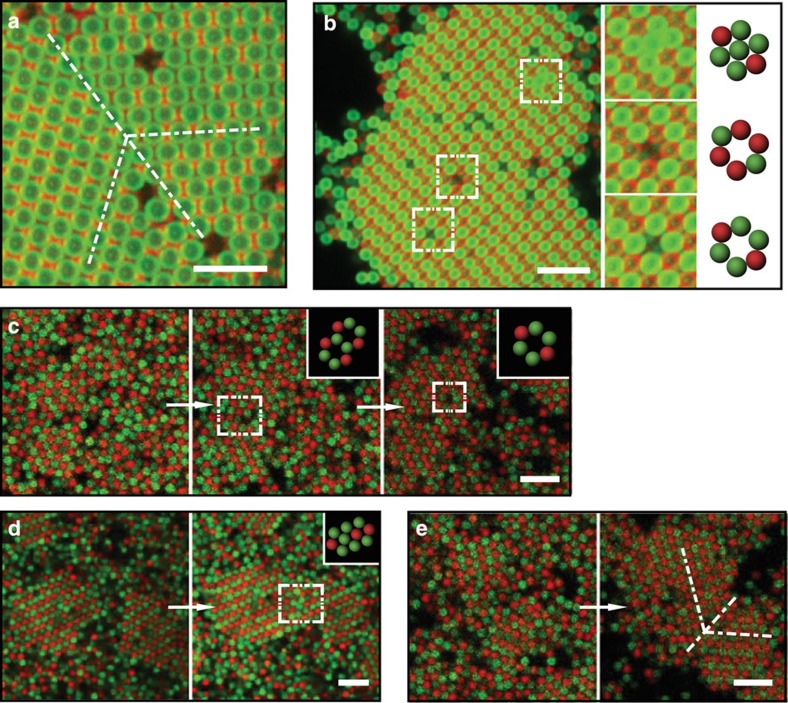
*In situ* observation of crystal defect formation. Confocal fluorescent and schematic images showing (**a**) a grain boundary in the 110 plane of a Cs_6_C_60_ crystal and (**b**) antisite as well as vacancy defects in a CsCl crystal. (**c**–**e**) Snapshots from videos showing the formation of crystal defects. (**c**) A three-particle vacancy defect is obtained at the intersection between two crystals and a single-particle vacancy defect forms as the crystal grows. (**d**) A green particle is trapped in a mismatched location when two crystals merge, leading to the formation of an antisite defect. (**e**) Grain boundaries form when two crystals merge, while their orientations are not aligned. See also Supplementary Movies 7 and 8. Scale bar, 5 μm.

We observe different kinds of defects in our crystals, including vacancy defects, antisite defects and grain boundaries. Figure 6a shows a twin boundary on the 110 plane of a Cs_6_C_60_ crystal, while Fig. 6b shows a variety of vacancy defects and an antisite defect. To observe the formation of these various defects, we follow the crystallization of fluorescent TPM particles using confocal microscopy (Supplementary Movie 7). We find that single-particle vacancy defects form as the crystals grow, while larger vacancy defects usually form when two crystals merge with their crystalline axes aligned (Fig. 6c). Antisite defects form when a particle is trapped in the ‘wrong' site during crystal merging or rapid crystal growth (Supplementary Movie 7). In Fig. 6d and Supplementary Movie 8, we show a case where a green particle present at the junction between two merging crystals is trapped in the wrong position, finding no suitable adhesion point. Grain boundaries develop when two crystals approach each other with misaligned crystalline axes, and are often accompanied by vacancy defects. Figure 6e shows the formation of grain boundaries in a CsCl lattice.

## Discussion

The ability of DNA-bound particles to diffuse and anneal means that DNA-coated colloids can surmount kinetic barriers and find pathways to form the structures they have been programmed to create. This opens up the study of self-assembly and defect formation *in situ* using conventional optical microscopy, providing an attractive model platform to study the self-assembly of particulate systems.

Moreover, using colloidal building blocks where the DNA coating is much thinner than the particle size makes possible a new materials science in which particles, and not DNA, constitute the majority component of the structure. Thus, DNA becomes a structure-directing glue for putting together different materials. Extending these techniques to make not only the binary crystals illustrated here, but to make much more complex structures out of different materials—plastics, inorganics, metals and semiconductors—is well within reach. In fact, we have also prepared polystyrene, poly(methylmethacrylate) and silica colloids with smooth surfaces and azide functional groups. High areal density ssDNA can be similarly grafted onto these particles using SPAAC; single component and heterogeneous binary colloidal crystals have been made from these materials.

Because these techniques can achieve areal densities of ssDNA exceeding 10^5^ ssDNAs per micrometre-sized particle, far in excess of the 10^4^ ssDNA required for particles to diffuse and anneal, different particles can be coated with many different ssDNA sticky-end codes, which should facilitate the programmed assembly of structures much more complex than the binary crystals demonstrated here^8^,^34^. The ability for DNA-bound particles to anneal should also facilitate assembling patchy colloidal particles into more open complex colloidal architectures^35^.

## Methods

### Microspheres fabrication

Particles possessing azide anchors for covalent DNA attachment are fabricated by copolymerizing TPM with 3-chloro-2-hydroxypropyl methacrylate (CHPMA) followed by azide substitution of the chlorine groups. Typically, 200 μl of TPM is added to 20 ml of aqueous solution containing ammonium hydroxide (1% w/w). The reaction is allowed to stir for 4 h at room temperature, producing monodisperse TPM emulsions. Then, 40 μl of CHPMA is added, which diffuses into the TPM emulsion droplets. For the sphere used in confocal videotaping, 5 mg Coumarin-modified styrene monomer or Rhodamine-modified methacrylate monomer is added with CHPMA. After 30 min, 5 ml of an aqueous solution of SDS (5% w/w) is introduced. Ten minutes later, 10 mg of azobis(isobutyronitrile) (AIBN) is added and the reaction mixture is allowed to stir for another 20 min before the temperature is raised to 80 °C. Thermal degradation of AIBN initiates the polymerization, generating the chlorine-functionalized particles. To make particles with rough surfaces, CHPMA and AIBN are premixed with TPM before hydrolysis and condensation. The emulsion is directly solidified without adding SDS to stabilize the emulsion. The resulting particles are purified by repeated centrifugation/redispersion and finally dispersed in 20 ml of an aqueous solution of Pluronic F127 (0.2% w/w) containing 500 mg of sodium azide (NaN_3_) and catalytic amount of potassium iodine (KI). The suspension is then heated at 70 °C for 12 h, yielding azide functionalized TPM particles. The particles are washed and stored in deionized water for further usage. Varying the TPM amount (CHPMA is kept at 20% v/v to TPM), particles of different sizes (*d*=0.5–3.5 μm) are obtained with low-size distribution (<5%; Supplementary Fig. 1a,b).

### DNA functionalization

Single-stranded oligonucleotides with sticky ends (Integrated DNA Technologies USA) are used in this study. 5′-Amino-DNAs are purchased and the amine groups are converted to a dibenzyl cyclooctane (DBCO) group by treating the DNA with DBCO-sulfo-NHS (Click Chemistry Tool) in PBS (10 mM, pH 7.4, 100 mM salt, same below). The DNA is also internally fluorescent labelled with Cy3 (emission maximum 564) or Cy5 (emission maximum 668), respectively. Both palindrome (P) and complementary (A/B) DNA are used, with the length of sticky end containing four or eight bases. The sequences are:

A4: 5′-/DBCO/(T)_20_-Cy3-(T)_41_-GCAG-3′

B4: 5′-/DBCO/(T)_20_-Cy5-(T)_41_-CTGC-3′

P4: 5′-/DBCO/(T)_20_-Cy5-(T)_41_-CGCG-3′

P8: 5′-/DBCO/(T)_20_-Cy5-(T)_41_-CGTATACG-3′

In a typical DNA grafting experiment, azide functionalized particles are first dispersed in 400 μl of PBS containing Triton-X-100 (0.1% w/w) with a particle concentration of 0.1% w/w. Then, 20 μl of DBCO-DNA (100 μM) is added to the particle suspension and the reaction mixture is stirred at 55 °C for 24 h, yielding the DNA-functionalized particles. The particles are washed and stored in PBS containing 1% w/w Pluronic F127 for the self-assembly experiments.

### Flow cytometry

We use flow cytometry to quantify the number of DNA strands functionalized per particle. Cy5-labelled microsphere are used as cytometry standard (Quantum Cy5 MESF, Bangs Laboratories Inc.). Using the provided molecules of equivalent soluble fluorochromes (MESF), a calibration curve is constructed, based on which the measured fluorescent intensity data for each of DNA-coated particle sample is converted to an approximate number of DNA grafted on each particle. Flow cytometry experiments are carried out using a BD LSRII HTS cytometer. Particle samples are dispersed in PBS with Pluronic F127 (1% w/w).

### Diffusion experiment

To investigate the mobility of bound DNA-coated colloids, we affix 2.0-μm DNA-coated colloidal particles to a glass microscope slide by embedding them in a thin polystyrene film spin coated on the slide (Supplementary Fig. 4). The slide forms the one side of a cell, which is subsequently filled with a very dilute suspension of 1.0-μm colloidal particles coated with DNA strands complementary to those on the immobilized 2.0-μm particles. The sample is heated above the melting temperature and then cooled below the melting temperature. After a period of time, a free 1.0-μm DNA-coated colloidal particle binds to an immobilized 2.0-μm particle attached to the substrate. We then track the motion of the 1.0-μm particle as it diffuses on the sphere for 6 min at 5 frames s^−1^.

### Measurements of particle roughness

The roughness of the particle surfaces was measured with a tapping mode atomic force microscopy in air. Results of typical scans of a smooth (left plots) and a rough particle (right plots) are shown in Supplementary Fig. 1d.

### Self-assembly

For the self-assembly studies, the particles of interest were combined, mixed according to the stoichiometry of the target crystalline structure and transferred to a glass capillary tube (2 mm × 100 μm × 10 cm). The capillary tube was pretreated with oxygen plasma for 1 min and exposed to hexamethyldisilazane vapour to render it hydrophobic to prevent DNA-coated colloids from sticking. After adding the sample, the capillary tube is sealed and attached to a microscope glass slide using wax. The slide is then mounted on a homemade microscope thermal stage with the ability to create a temperature gradient. For crystal growth, the sample is first heated above the melting temperature to melt any aggregates and then quenched to different temperatures below *T*_m_ and held constant.

Crystals are observed to form only when the number ratio of particles mixed is near the stoichiometry of the target crystalline structure. For example, when making Cs_6_C_60_ crystals, the number ratio of 0.54-μm particles to 1.5-μm particles is kept around 6:1. Slight changes in this ratio still result in the same crystal structure but with a different amount of crystal vacancy defects. For instance, when the ratio is 4:1 (insufficient 0.54-μm particles), we find many vacancies for the smaller particles. When the ratio is increased to 8:1, very few vacancies are found. For greater degrees of non-stoichiometric preparations, crystals are not observed.

### Microscopy

Bright-field optical images and videos were obtained using a Nikon TE300 microscope equipped with a CCD camera. Fluorescent images and videos were taken using a Leica SP8 confocal fluorescence microscope. Some of the microscope images and videos were digitally post-processed to improve brightness and contrast.

## Additional information

**How to cite this article:** Wang, Y. *et al.* Crystallization of DNA-coated colloids. *Nat. Commun.* 6:7253 doi: 10.1038/ncomms8253 (2015).

## Supplementary Material

Supplementary Information Supplementary Figures 1-5, Supplementary Table 1, Supplementary Note 1 and Supplementary Reference

Supplementary Movie 1 Crystallization of 1.0 μm P particles. Crystallization kinetics for 1.0 μm P (palindrome) particles held at 45.0°C immediately after a rapid quench from above the melting temperature Tm = 46.5°C. Particles spontaneously nucleate within five minutes after the quench and form FCC crystals that encompass nearly the entire sample within 60 minutes. The Movie is played at 20 frame/s, 200 × real time. Time stamp on this and all Movies is hours : minutes : seconds.

Supplementary Movie 2 Particle rolls around. The mobility of DNA bonded particles is investigated at three different temperatures. The tracks of a small particle (1.0 μm in diameter) are recorded as it rolls on a larger particle (2.0 μm in diameter) that is immobilized on a glass substrate. The small particle diffuses more rapidly for shallow quench (the temperature is higher and closer to Tm). The Movie was acquired at 5 frame/s and is played at 50 frame/s.

Supplementary Movie 3 Crystal formation at 28.3°C. This Movie shows the formation of CsCl colloidal crystals at 28.3°C (T-Tm = -0.6°C) using 0.54 μm A and B particles. The crystallization proceeds by nucleation and growth. The first stable crystal nucleates around 45 minutes and grows, along with several others that nucleate subsequently, into 5 large crystals in approximately 180 minutes. The image of the first growing crystal is shifted to the middle of viewing area at 01:18:30. The Movie is played at 20 frame/s, 600 × real time.

Supplementary Movie 4 Crystal formation at 27.9°C. This Movie shows the formation of a CsCl colloidal crystal at 27.9°C (T-Tm = -1.0°C) using 0.54 μm A and B particles. The crystallization proceeds by nucleation and growth. The first crystals nucleate around 15 minutes and subsequently grow into 14 large crystals in approximately 95 minutes. The Movie is played at 20 frame/s, 400 × real time.

Supplementary Movie 5 Crystal formation at 27.5°C. This Movie shows the formation of CsCl colloidal crystal at 27.5°C (T-Tm = -1.4°C) using 0.54 μm A and B particles. It takes only 10 minutes for crystals to nucleate and 45 minutes for most of the particles (>90%) to crystallize. The time required is significantly shorter compared to both higher temperature and lower temperature. The Movie is played at 20 frame/s, 200 × real time.

Supplementary Movie 6 Crystal formation at 27.0°C. This Movie shows the formation of CsCl colloidal crystal at 27.0°C (T-Tm = -1.9°C) using 0.54 μm A and B particles. The crystal formation proceeds by a two-stage process in which a dense amorphous aggregate forms very rapidly, followed by slow crystallization via local rearrangement of particles. Crystals nucleate around 20 minutes and grow to about 110 small crystals in approximately 210 minutes. The Movie is played at 20 frame/s, 800 × real time.

Supplementary Movie 7 Defect formation. This confocal microscope Movie shows the crystallization of a CsCl colloidal crystal, illustrating the formation of various defects. Fluorescent 1.0 μm A and B particles whose fluorescence originates from copolymerized Coumarin and Rodamine dye (see Methods) are used to avoid bleaching during the extended laser exposure. Single particle vacancy defects, multiple-site vacancy defects, antisite defects, and grain boundaries are shown at a single-particle resolution. A Tokai Hit stage top incubator is used to heat the sample. The Movie played at 20 frame/s, 800 × real time.

Supplementary Movie 8 Antisite defect formation. This confocal microscope Movie shows the formation of an antisite defect. This defect develops when two aligned crystals merge. Particle types and experiment condition are the same as those described in Supplementary Movie 7. The Movie is played at 20 frame/s, 160 × real time.
