# Supplementary material for: Crystallization of DNA-coated colloids
Source: Nat Commun. 2015 Jun 16;6:7253. doi: 10.1038/ncomms8253 (PMC4490366; doi:10.1038/ncomms8253)
Supplement: Supplementary Information — Supplementary Figures 1-5, Supplementary Table 1, Supplementary Note 1 and Supplementary Reference [file ncomms8253-s1.pdf]

Supplementary Figures

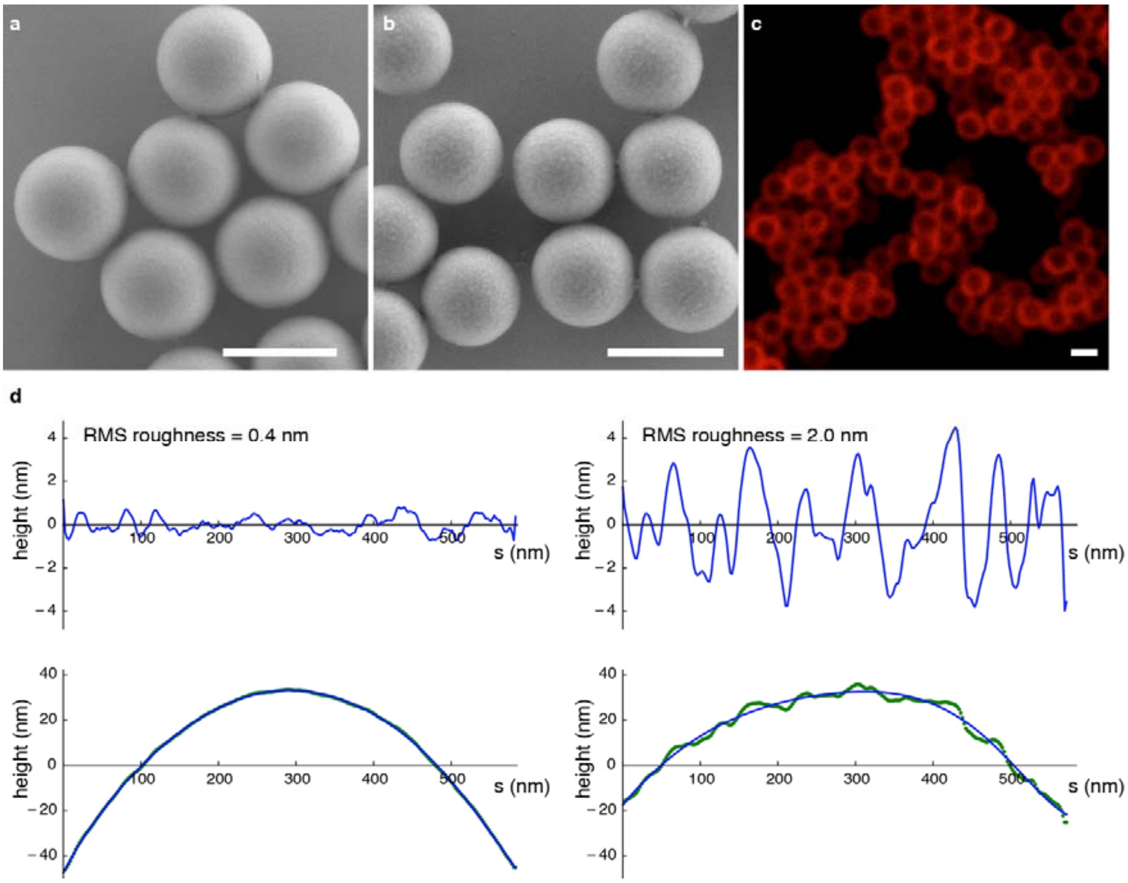

Supplementary Figure 1 | TPM particles. Scanning electron micrograph showing monodisperse colloids with **a**, smooth surfaces, **b**, rough surface. **c**, Confocal fluorescent image showing colloids with a bright florescent corona, indicating a dense and uniform DNA coating. **d**, Typical atomic force microscopy (AFM) scans of a smooth (left plots) and a rough particle (right plots). The particle roughness is extracted by calculating the root mean square (RMS) deviations from a perfectly smooth surface, shown by the blue lines. Scale bars, 1 µm.

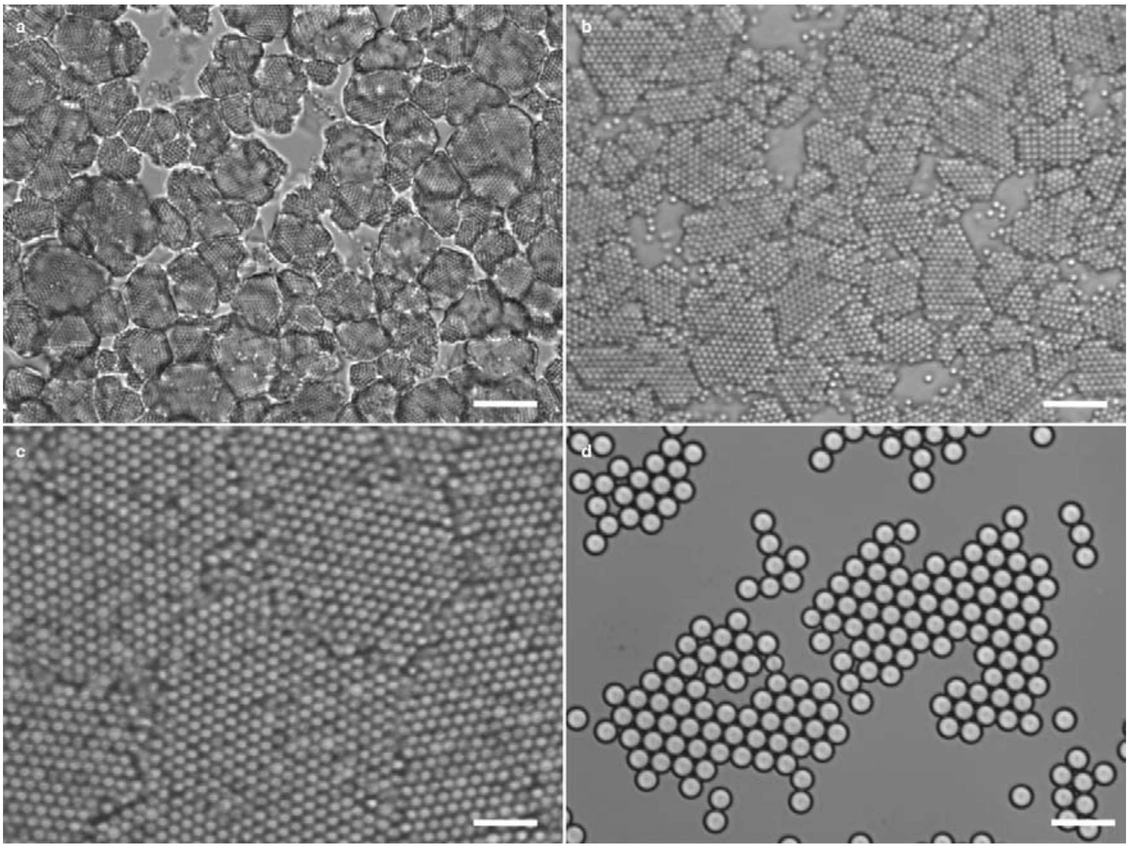

Supplementary Figure 2 | Colloidal crystals assembled from particles of various sizes. Bright-field microscope images showing FCC crystals assembled from **a**, 0.54-µm, **b**, 1.0-µm, **c**, 2.0-µm, and **d**, 3.5-µm particles coated with ssDNA bearing palindrome sticky ends. Scale bars, 10 µm.

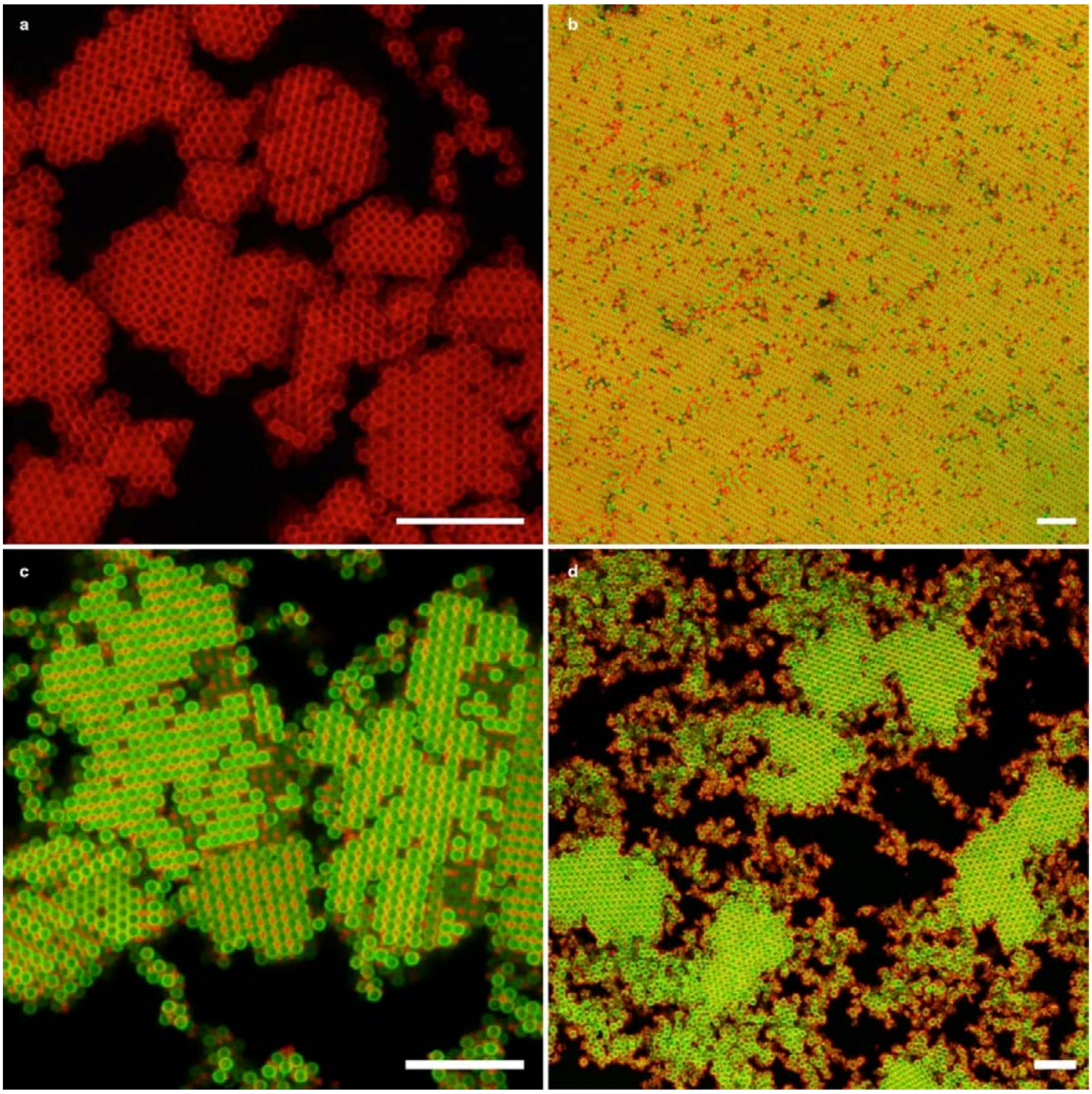

Supplementary Figure 3 | Colloidal crystals assembled from DNA coated colloids. Confocal fluorescent images showing a "full field of view" of various types of colloidal crystals. **a**, An FCC lattice assembled from 1.0-μm P particles, verified by observations of several different crystalline planes. **b**, A single crystal AB lattice (isostructural to CsCl) assembled from 1.0-μm particles with complementary DNA sticky ends. The sample is annealed at shallow quench for 50 hours. The green fluorescent particles are

coated with ssDNA with A6 sticky ends (TGCGGT) and red fluorescent particles are coated ssDNA with B6 sticky ends (ACCGCA). **c**, An AB_2 crystal (isostructural to AlB_2) is obtained using 1.0-µm A and 0.54-µm B particles. **d**, An AB_6 crystal lattice (isostructural to Cs_6C_{60}) assembles from 1.5-µm A and 0.54-µm B particles. Scale bars, 10 µm.

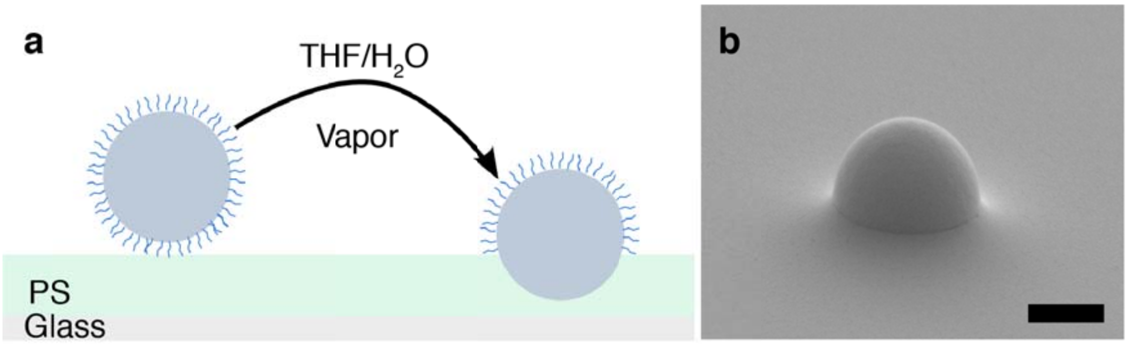

Supplementary Figure 4 | Immobilized DNA-coated colloids. **a**, Schematic illustration showing the process to affix a 2.0-µm A particle on a glass microscope slide. The DNA coated A particle is partially embedded in a thin polystyrene film spin-coated on the slide, with the assistance of THF/water vapor to plasticize the polymer film and let the particles sink. **b**, Scanning electron micrograph of the embedded particle. Roughly one hemisphere of the A particle is available for DNA binding.

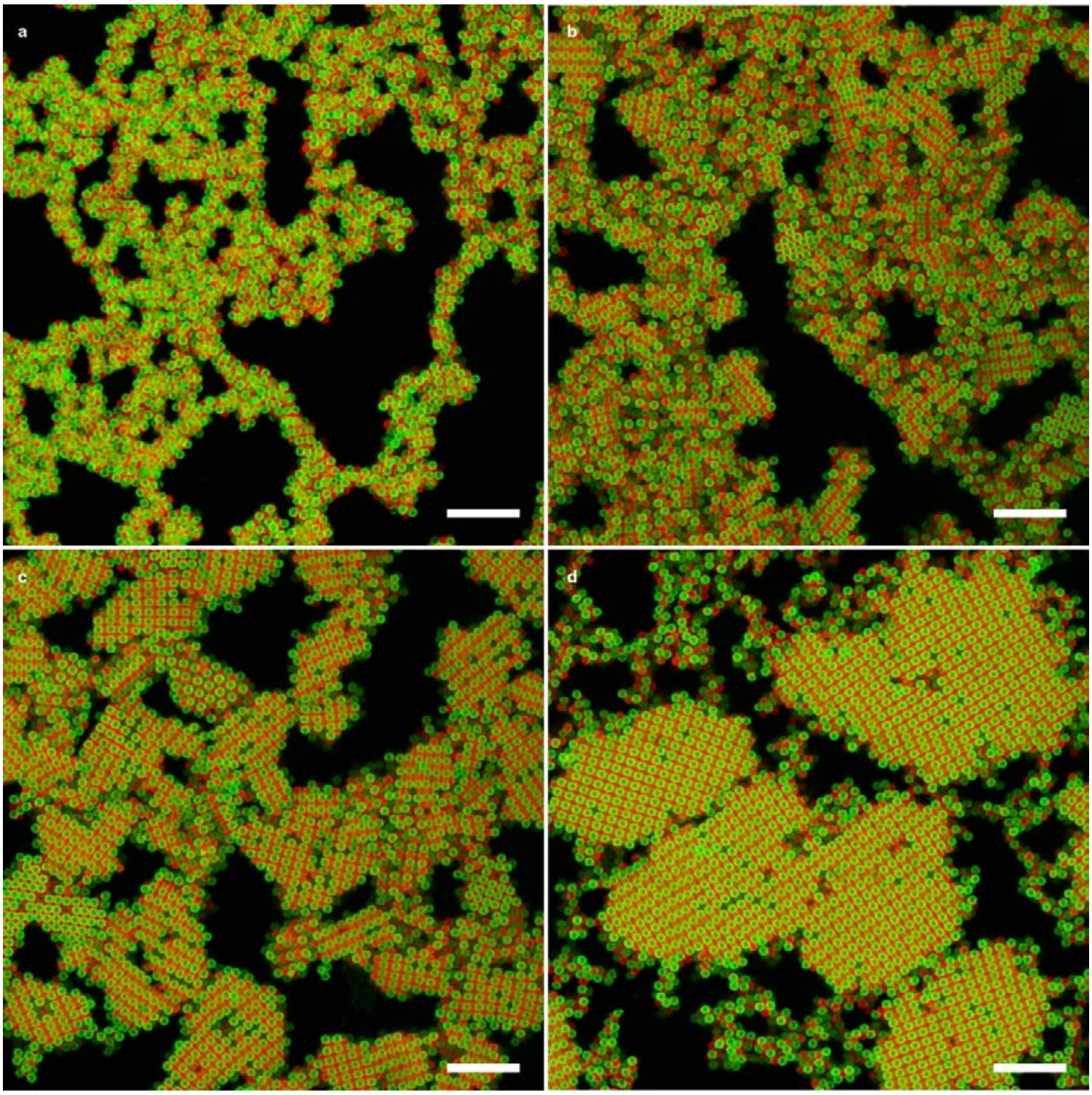

Supplementary Figure 5 | Temperature-dependent crystal sizes. Crystals assembled from 1.0-μm A and B particles at **a**, 26.0 °C, **b**, 27.7 °C, **c**, 28.6 °C, and **d**, 29.3 °C, illustrating the effect of annealing temperature on the size and conversion percentage of colloidal crystals. The sample is annealed for ten hours. Scale bars, 10 μm.

Supplementary Table

Temperature (°C)	27	27.5	27.9	28.3
Aggregation time (min)	3.1±0.2	3.7±0.2	14±1	45±2

Supplementary Table 1 | Aggregation time of DNA coated colloids at various temperatures. The time required for DNA-coated colloids to form stable or metastable clusters after a rapid quench from above the melting temperature T_m = 28.7°C.

Supplementary Note 1

Single stranded DNA (ssDNA) consisting of 61 bases is grafted to the surfaces of the colloidal particles. The distance between nucleotides for ssDNA is b_0 = 0.63 nm[26] which gives a contour length of L = 38.4 nm. The persistence length L_p = 2.5 nm for 100 mM NaCl[26]. In this case the mean squared end-to-end length given by the worm-like chain model (Supplementary Equation 1) is

$$R^2 = 2LL_p\left[1-\frac{L_p}{L}\left(1-e^{-L/L_p}\right)\right], \tag{1}$$

which gives R = 13 nm. The areal density of DNA is measured by flow cytometry to be σ = 1 ssDNA/27 nm^2, which corresponds to a mean distance of d = 5.2 nm between grafting points. Because $d < R$, the grafted chains are stretched. For stretched chains, Milner, Witten, and Cates[27] give the following expression (Supplementary Equation 2) for the distribution of end heights

$$n(z) = \frac{\pi^2 z\sqrt{h^2-z^2}}{2w(L/L_p)^3}, \tag{2}$$

where z is the distance from the colloid surface, w is the excluded volume parameter, and $h = (L/L_p)(12\sigma w/\pi^2)^{1/3}$. Taking the excluded volume to be $w \approx \lambda_D L_p^2$, where λ_D is the Debye length, about 1 nm at 100 mM NaCl, the height h of the ssDNA brush is about 13-17 nm, depending on the precise value of w. Recent AFM measurements[1] of brush heights at comparable areal density of 44-base ssDNA give a height of approximately 8 nm, which extrapolating to our 61-base ssDNA gives a height of 11 nm, somewhat smaller but consistent with the above estimate given the uncertainties in the experimental parameters and approximate nature of the models.

The extent of the lateral reach of the DNA ends should be Gaussian distributed with the root mean square distance given by $R_l \approx \sqrt{2LL_p} = 14$ nm, as there is no stretching of the chains in the lateral direction (in contrast to the vertical direction). Figure 3h plots the mean distance d between active sticky ends and reveals that the particles fail to completely crystallize when d exceeds R_l. In this limit, ssDNA strands with sticky ends cease to be able to reach more than one ssDNA sticky end on the particle to which it binds, which would be expected to suppress bound particles from rolling on each other.

Supplementary Reference

1 Nkoua Ngavouka, M. D., Bosco, A., Casalis, L. & Parisse, P. Determination of average internucleotide distance in variable density ssdna nanobrushes in the presence of different cations species. *Macromolecules* **47**, 8748-8753 (2014).
